# Hyperbaric Oxygen Therapy in the Management of Refractory Perianal Crohn’s Disease

**DOI:** 10.3390/jcm14196843

**Published:** 2025-09-27

**Authors:** Roy Hajjar, Katherine A. Bews, Ahmed Amine Alaoui, Sidrah Khan, Lauren Gleason, Emilio Sanchez, Ian S. Reynolds, Sunanda V. Kane, William R. Perry, Kellie L. Mathis, Nicholas P. McKenna

**Affiliations:** 1 Division of Colon and Rectal Surgery, Mayo Clinic, Rochester, MN 55904, USA; 2 Robert D. and Patricia E. Kern Center for the Science of Health Care Delivery, Mayo Clinic, Rochester, MN 55904, USA; 3 Division of Gastroenterology and Hepatology, Mayo Clinic, Rochester, MN 55904, USA

**Keywords:** Crohn’s disease, perianal Crohn’s disease, hyperbaric oxygen therapy, perianal fistula

## Abstract

**Background:** Crohn’s disease (CD) is an inflammatory bowel disease (IBD) that is prevalent worldwide. It can affect any segment of the gastrointestinal tract, from the mouth to the anus. When CD affects the anus, perianal fistulizing disease develops. The management of perianal CD is challenging and may require morbid surgery when there is no response to medical therapy. The emergence of novel biologic therapies, namely tumor necrosis alpha (TNF-α) inhibitors, has proven to provide long-term relief and prevent disease-related complications. Perianal CD is, however, refractory or recurrent in up to 80% of patients. One of the reported options to manage perianal CD is hyperbaric oxygen therapy (HBOT), which aims at increasing tissue oxygen saturation in an attempt to promote repair and reverse local inflammation. Data on this approach is scant. **Methods**: A retrospective review was performed to identify patients with CD at the Mayo Clinic in Rochester who underwent HBOT for perianal disease between 2014 and 2023. Demographic and clinical data were reviewed, including the history of the disease, concomitant medical and surgical therapy and the need for fecal diversion. The HBOT regimen, including the number of sessions and clinical response, were reviewed. **Results**: Six patients aged from 19 to 60 years underwent HBOT for perianal CD. Two patients had a history of total proctocolectomy with ileal-anal pouch anastomosis (IPAA). All patients except one were on immunosuppressive medication including biologic agents. Four patients had fecal diversion with an ileostomy or colostomy. Patients received between 10 and 40 sessions of HBOT. Four patients reported symptomatic improvement. On physical examination and/or imaging assessment, improvement was noted in one patient. Progression of the perianal disease was noted in all other patients, with all except one requiring an operation in the following year. **Conclusions**: HBOT may provide symptomatic relief in some patients with refractory perianal CD, but data on its long-term efficacy remains limited.

## 1. Introduction

Initially described in the 20th century, Crohn’s disease (CD) is a globally prevalent autoimmune condition with increasing incidence that may affect any segment of the gastrointestinal tract from the mouth to the anus [1,2,3,4]. The pathogenesis is believed to be due to a combination of genetic factors, environmental exposures and dysregulation of the immune response in the gastrointestinal mucosa [5,6,7]. This leads to sustained inflammation mainly affecting the terminal ileum, or the rest of the small or large bowel. After initially starting as transmural inflammation, over time, the disease usually follows one of two distinct phenotypes, stricturing or penetrating [8,9,10]. In the stricturing form of the disease, chronic inflammation and fibrosis lead to the obliteration of the intestinal lumen, and to intestinal obstruction in the most severe forms [11]. In penetrating disease, also known as fistulizing disease, chronic transmural inflammation leads to the extension of inflammation beyond the bowel wall, and to the formation of fistula and abscesses [12,13]. When penetrating CD affects the anus, perianal fistulizing disease develops [13]. The management of CD has greatly evolved over the last decades. While initial therapy relied on anti-inflammatory drugs including 5-aminosalicylates, steroids and immunomodulators, including thiopurines and methotrexate with poor long-term control, the emergence of novel therapies has proven to provide long-term relief and prevent disease-related complications [13,14]. The wide emergence of biologic therapy in the early 21st century, specifically tumor necrosis alpha (TNF-α) inhibitors, changed the paradigm of treatment, and proved to be a significant breakthrough in long-term disease control [15,16].

Perianal disease has been reported in approximately 20% of patients with CD [17]. It consists mostly of fistulas, abscesses and fissures [18]. Due to the high density of bacteria locally, inflammation is believed to become more refractory, and is associated with significant symptoms and poorer quality of life [19]. In the severe forms of perianal disease, surgery may become the only option, requiring extensive abdominoperineal resection [20]. The use of biologics in patients with perianal CD may provide significant control of perianal inflammation, and sometimes lead to the resolution of perianal symptoms [20]. It is therefore recommended as a first-line therapy in patients with perianal CD, with surgery used for abscess drainage and to control symptomatic fistula tracts with setons [21,22,23]. Definitive surgical management therapy for CD-associated fistulas is usually not recommended in most patients due to chronic inflammation being the underlying factor in its pathogenesis.

Despite advances in medical therapy and biologics, perianal CD is refractory or recurrent in up to 80% of patients and remains, therefore, a challenging presentation [23,24]. One of the reported options to manage perianal CD is hyperbaric oxygen therapy (HBOT) [25]. HBOT has been described in this setting beginning in the 1980s, where its use provided symptomatic relief in a patient with severe and refractory perianal CD [26]. The use of this therapy aims at targeting tissue oxygenation and hypoxia, and increasing tissue oxygen saturation in an attempt to promote repair and reverse local inflammation [27]. Its role in the treatment of perianal CD remains undefined.

We report in this manuscript our experience with this modality at Mayo Clinic in Rochester, and review previous reports on its use in the setting of refractory perianal CD to assess its usefulness in this patient population.

## 2. Methods

Institutional databases were reviewed for cases of patients who received HBOT for perianal CD at Mayo Clinic in Rochester between 2014 and 2023. All patients with CD (international classification of disease (ICD) 9: 555.x and ICD 10: K50.x) or ulcerative colitis (UC) (ICD 9: 556.x and ICD 10: K51.x) and a current procedure terminology (CPT) 99183 or a diagnosis code of 93.95 (ICD 9), 5A05121 (ICD 10) or 5A05221 (ICD 10), which indicate receipt of HBOT, were identified. A retrospective review was performed to identify patients with CD who underwent HBOT for perianal disease. Charts of patients with UC were also reviewed to identify those with a subsequent diagnosis change to CD.

Demographic and clinical data were reviewed, including the history of the disease, concomitant medical and surgical therapy, and the need for fecal diversion. The HBOT regimen, including the number of sessions and clinical response based on the clinician’s assessment and imaging studies, were reviewed. Descriptive statistics were completed.

## 3. Results

Six patients aged from 19 to 60 years underwent HBOT for perianal CD. Two patients had a history of total proctocolectomy with ileal-anal pouch anastomosis (IPAA). All patients except one were on immunosuppressive medication including biologic agents. Four patients had fecal diversion with an ileostomy or colostomy. Patients received between 10 and 40 sessions of HBOT. Four patients reported symptomatic improvement. On physical examination and/or imaging assessment, improvement was noted in one patient. Progression of the perianal disease was noted in all other patients, with all except one requiring an operation in the following year. Full clinical and demographic data are depicted in Table 1.

## 4. Discussion

Perianal CD afflicts a significant portion of patients with CD. The current standard of care is medical therapy with biologic agents that are associated with a positive response in more than half of the patients, and surgery for patients with perianal abscesses [28]. As the inflammation becomes chronic, cycles of active inflammation and partial recovery lead to chronic changes including fistulas and strictures that no longer respond to medical therapy, and that may require surgical treatment to palliate symptoms. HBOT has been proposed as an adjunct to standard therapy in the management of refractory perianal CD, but there is limited data in support of it. The data from this case series indicates that while this therapeutic modality may provide symptomatic relief, it may not lead to long-term sustained remission.

One of the underlying hypotheses behind anorectal CD is that it is secondary to tissue hypoxia. Hypoxia contributes to poor tissue perfusion and sustained local inflammation. Persistent inflammation contributes in turn to a chronic wound phenotype where the inflammatory phase fails to progress to the healing proliferative phase required for proper healing and sealing of the fistula tract. This complex process is believed to be due to the constant inflammatory signals that prevent pro-proliferative cytokines and chemokines from promoting a healing phenotype [29]. Achieving adequate oxygen perfusion of the tissues is therefore believed to contribute not only to decreased inflammation, but also to improved healing of the anal canal and perianal region [30,31,32,33]. Nonetheless, there is a lack of data on the efficacy of HBOT in patients with CD.

The role of hypoxia in IBD is complex and involves the regulation of several key pathways implicated in epithelial homeostasis, with possible involvement of the gut microbiome [30,33,34]. The premises underlying the use of HBOT in CD are that hypoxia is linked to intestinal inflammation, and that reversing epithelial hypoxia may promote an anti-inflammatory phenotype in the gut [31,32,35]. However, there is data that also suggests that hypoxia may actually ameliorate intestinal inflammation [36,37]. These contradictory findings further complicate the use of this therapy in patients with IBD, and indicate that the use of HBOT may not be feasible and efficient systematically in all patients with perianal CD. Fortunately, HBOT remains safe overall. The major side effects include ear barotrauma, sinus pain or pressure and temporary myopia that usually resolve with stopping the treatment [38,39].

Previous data on the use of HBOT in patients with perianal CD is scant, with a few manuscripts reporting conflictual data over a period of several decades (Table 2). All reports described either symptomatic relief or healing of the fistulizing disease. All patients also received concomitant medical therapy, and many were reported to have had surgery as well. Overall, these papers report improvement or full recovery on short-term follow-up, but data on long-term follow-up and recurrence are lacking. The findings here support symptomatic improvement without durable long-term healing. There may be a selection bias present in these patients, with only patients with the most severe perianal CD phenotype referred for HBOT.

With the emergence of more efficient inflammation-targeting agents, namely monoclonal antibodies, HBOT may become a valuable adjunct in selected patients. Previous data suggest that this approach has a limited but potential real benefit in some patients. It does not seem however to provide on its own long-term sustained effects. This may be due to the chronic nature of the disease and the multifactorial and complex inflammatory process. Targeting tissue hypoxia may be beneficial but not sufficient to achieve significant suppression of local inflammation. One potential option may be to consider HBOT as a recurrent treatment, with cyclic sessions over a long period of time. Its role in the setting of fecal diversion may possibly also contribute potentially to a higher remission rate, as a continuous fecal stream may exacerbate inflammatory stimuli and prevent recovery. These approaches need, however, further assessment in clinical practice to determine their impact.

This case series has several limitations. First, this is a descriptive report where the low number and heterogeneity of the patients, clinical presentations and therapeutic regimens make the assessment of the impact of HBOT and data generalizability challenging. Multicenter collaborations are required to overcome this major limitation and strengthen the findings. Randomized controlled trials including standardized symptom assessment using validated scales would help draw stronger conclusions. Second, the cases were reviewed over a long period of time, over which medical therapy for IBD evolved significantly with the introduction of several biologics, potentially confounding the effect of other therapies. Finally, the differences in the HBOT regimen, the inability to systematically assess side effects and the heterogeneity of baseline CD treatment make the evaluation of HBOT on disease recovery or progression difficult.

In summary, our data suggest that HBOT seems to provide temporary symptomatic relief in patients with refractory perianal CD, although disease progression and the need for other therapies remain common. Data on its efficacy is scarce, however, and further data, ideally from randomized trials, is required to determine its role, potentially, as an adjunct to other therapies in this patient population.

## Figures and Tables

**Table 1:** Patients with perianal Crohn’s disease receiving hyperbaric oxygen therapy (N = 6).

Age (Years)	Sex	History	Therapies	Diversion	HBOT (Sessions)	Symptoms	Physical or Imaging Assessment	Disease Progression
24	F	Presumed UC with IPAA, fistulizing perianal CD requiring EUAs	Sulfasalazine, infliximab, adalimumab, budesonide, steroids, 6-MP, antibiotics, vedolizumab, ustekinumab	Yes (ileostomy)	20	Improvement	No significant improvement	Yes (EUA < 1 year)
43	M	Ileal and fistulizing perianal CD requiring EUAs	Azathioprine, ustekinumab, methotrexate	Yes (ileostomy)	20	Improvement	Improvement in MRI at 2 months	Yes (worsening disease on MRI after 15 months)
52	M	Fistulizing abdominal and perianal CD	Prednisone, methotrexate, ustekinumab	No	40	No improvement	No improvement	Yes (proctocolectomy < 1 year)
19	F	Abdominal and fistulizing perianal CD	Adalimumab and ustekinumab	No	20	Improvement	No improvement (on imaging)	Yes (EUA < 1 year)
60	F	Abdominal and fistulizing perianal CD	Infliximab, prednisone, sulfasalazine	Yes (colostomy)	10	Improvement	No improvement (on imaging)	Yes (EUA < 1 year)
29	F	IPAA, fistulizing perianal disease	Recurrent EUAs	Yes (ileostomy)	40	No improvement	No improvement	Yes (IPAA excision < 1 year)

HBOT, hyperbaric oxygen therapy; EUA, examination under anesthesia; CD, Crohn’s disease; UC, ulcerative colitis; IPAA, ileal-anal pouch anastomosis; MRI, magnetic resonance imaging.

**Table 2:** Previous reports on the use of hyperbaric oxygen therapy in patients with perianal Crohn’s disease.

Year	Authors	Country	Journal	Type	N	Age (Years), Median	HBOT Regimen	Adjuncts	Outcome
2025	Mulders et al. [40]	The Netherlands	Journal of Crohn’s and Colitis	Abstract	16	32.5	40 sessions	Seton, surgery (closure of internal opening), optimized medical therapy	Clinical remission (no draining opening) 70% and PDAI ≤ 4 at week 52, MRI healing 40% at week 52. Improved quality of life
2024	Piotrowicz et al. [41]	Poland	Gastroenterology Review	Article	11	30.6 ^#^	Up to 30 sessions	Medical therapy, and surgery	54.5% clinical remission rate at 9 months
2024	Leong et al. [42]	Singapore	Cureus	Case report	1	21	60 sessions	Medical therapy	Healing of the fistula at 16 weeks after HBOT, PDAI decrease from 12 to 3, recurrence of the fistula after 9 months
2024	Tome and Kane [25]	USA	Gastroenterology and Hepatology	Case report	1	19	20 sessions	Medical therapy, seton placement	Improved symptoms
2022	Lansdorp et al. [43]	The Netherlands	United European Gastroenterology Journal	Article	20	34	40 sessions	Medical therapy, seton removal after 30 sessions	Significant decrease in PDAI and modified Van Assche index at week 60
2021	Lansdorp et al. [44]	The Netherlands	Alimentary Pharmacology and Therapeutics	Article	20	34	40 sessions	Medical therapy, seton removal after 30 sessions	Significant decrease in PDAI and modified Van Assche index at week 16
2021	Feitosa et al. [45]	Brazil	Gastroenterology Research and Practice	Article	25 **	NA	10–86 sessions	Medical therapy (azathioprine, anti-TNF agents, steroids)	Healing rate of 80%
2020	Lansdorp et al. [46]	The Netherlands	United European Gastroenterology journal	Article	3	34	40 sessions	Medical therapy, surgery	Healing in one patient, one patient with relapse at 3 months, one patient with improvement
2017	Bosa et al. [47]	Italy	Digestive and Liver Disease	Abstract	1	16	14 sessions	Medical therapy (adalimumab, azathioprine), antibiotics, surgical drainage	Clinical improvement
2017	Piotrowicz et al. [48]	Poland	Journal of Crohn’s and Colitis	Abstract	7	28	30 sessions	Medical therapy (immunomodulatory and/or biologic therapy)	Out of seven patients, improved CDAI in five, decreased fecal calprotectin in six, and decreased C-reactive protein in two, regression of lesions on MRI in five patients
2016	Feitosa et al. [49]	Brazil	Acta Cirúrgica Brasileira	Article	15 **	38	30 sessions ***	Medical therapy, surgery	Healing in 65% of patients
2015	Agrawal et al. [50]	Australia	Future Science OA	Article	9	32	Up to 30 sessions	Medical therapy, anti-MAP (*Mycobacterium avium*), surgery	Clinical improvement in all patients
1995	Colombel et al. [51]	USA	Diseases of the Colon and Rectum	Article	10	30 ^#^	30 to 40 sessions	Medical therapy (e.g., azathioprine, 5-ASA), prior surgery	Six out of eight patients who completed at least 30 sessions had partial or complete healing
1994	Lavy et al. [52]	Israel	Journal of Clinical Gastroenterology	Article	10	38	Up to 60 sessions	Medical therapy (salicylates, corticosteroids, 6-mercaptopurine, antibiotics)	Complete healing in six patients, improvement in four patients
1990	Nelson et al. [53]	USA	Digestive Diseases and Sciences	Case report	1	NA	74 sessions	Medical therapy (steroids, antibiotics), surgery	Clinical improvement
1989	Brady et al. [26]	USA	Gastroenterology	Case report	1	48	130 sessions	Medical therapy, surgery	Clinical improvement

NA, not available; ** cases of perineal Crohn’s disease; *** median number of sessions; ^#^ Average age; N, number; HBOT, hyperbaric oxygen therapy; PDAI, perianal disease activity index.

## Data Availability

The original contributions presented in this study are included in the article. Further inquiries can be directed to the corresponding author.
